# Experience of a Strategy Including CYP2C19 Preemptive Genotyping Followed by Therapeutic Drug Monitoring of Voriconazole in Patients Undergoing Allogenic Hematopoietic Stem Cell Transplantation 

**DOI:** 10.3389/fphar.2021.717932

**Published:** 2021-10-20

**Authors:** Irene García-García, Irene Dapía, Jaime Montserrat, Lucía Martinez de Soto, David Bueno, Lucía Díaz, Javier Queiruga, Amelia Rodriguez Mariblanca, Pilar Guerra-García, Elena Ramirez, Jesus Frías, Antonio Pérez Martínez, Antonio J. Carcas-Sansuan, Alberto M. Borobia

**Affiliations:** ^1^ Clinical Pharmacology Department, IdiPAZ, La Paz University Hospital School of Medicine, Autonomous University of Madrid, Madrid, Spain; ^2^ Medical and Molecular Genetics Institute (INGEMM), La Paz University Hospital, Madrid, Spain; ^3^ Paediatric Haemato-oncology Department, University Hospital La Paz, Madrid, Spain

**Keywords:** voriconazole, pharmacogenetic, preemptive, therapeutic drug monitoring, CYP2C19

## Abstract

Many factors have been described to contribute to voriconazole (VCZ) interpatient variability in plasma concentrations, especially CYP2C19 genetic variability. In 2014, Hicks et al. presented data describing the correlation between VCZ plasma concentrations and CYP2C19 diplotypes in immunocompromised pediatric patients and utilized pharmacokinetic modeling to extrapolate a more suitable VCZ dose for each CYP2C19 diplotype. In 2017, in our hospital, a clinical protocol was developed for individualization of VCZ in immunocompromised patients based on preemptive genotyping of CYP2C19 and dosing proposed by Hicks et al., Clinical Pharmacogenetics Implementation Consortium (CPIC) clinical guidelines, and routine therapeutic drug monitoring (TDM). We made a retrospective review of a cohort of 28 immunocompromised pediatric patients receiving VCZ according to our protocol. CYP2C19 gene molecular analysis was preemptively performed using PharmArray^®^. Plasma trough concentrations were measured by immunoassay analysis until target concentrations (1–5.5 μg/ml) were reached. Sixteen patients (57.14%) achieved VCZ trough target concentrations in the first measure after the initial dose based on PGx. This figure is similar to estimations made by Hicks et al. in their simulation (60%). Subdividing by phenotype, our genotyping and TDM-combined strategy allow us to achieve target concentrations during treatment/prophylaxis in 90% of the CYP2C19 Normal Metabolizers (NM)/Intermediate Metabolizers (IM) and 100% of the Rapid Metabolizers (RM) and Ultrarapid Metabolizers (UM) of our cohort. We recommended modifications of the initial dose in 29% (*n* = 8) of the patients. In RM ≥12 years old, an increase of the initial dose resulted in 50% of these patients achieving target concentrations in the first measure after initial dose adjustment based only on PGx information. Our experience highlights the need to improve VCZ dose predictions in children and the potential of preemptive genotyping and TDM to this aim. We are conducting a multicenter, randomized clinical trial in patients with risk of aspergillosis in order to evaluate the effectiveness and efficiency of VCZ individualization: VORIGENIPHARM (EudraCT: 2019-000376-41).

## Introduction

Voriconazole (VCZ) is a second-generation triazole antifungal agent with broad-spectrum activity against a variety of fungal infections. It is indicated for the treatment of invasive aspergillosis, candidaemia in nonneutropenic patients, fluconazole-resistant invasive infections of *Candida*, and severe fungal infections of *Scedosporium* spp. and *Fusarium* spp. In the case of invasive aspergillosis, VCZ appears as first-line therapy in the treatment guidelines (Patterson et al., 2016). Additionally, VCZ is commonly used as a prophylaxis agent in immunocompromised patients, highly susceptible to invasive fungal infections (IFIs) (Hicks et al., 2014; Solano et al., 2017). VCZ is characterized by nonlinear pharmacokinetics and wide interpatient variability in serum concentrations, especially in pediatric population (Hicks et al., 2014; Boast et al., 2016), which is directly related to both VCZ efficacy and the occurrence of adverse drug reactions (ADRs) (Park et al., 2012). In this context, the early achievement of VCZ therapeutic plasma concentrations is essential in order to avoid hepatotoxicity and neurotoxicity (Park et al., 2012) without compromising VCZ antifungal activity.

Many factors have been described to contribute to this variability, especially *CYP2C19* genetic variability, age, drug interactions, and liver disease (Miyakis et al., 2010; Wang et al., 2014a). VCZ has an extensive hepatic metabolism mainly through CYP2C19 and a small contribution of CYP3A4, CYP2C9, and FMO3 (Whirl-Carrillo et al., 2012). It has been well reported that the *CYP2C19* genotype is related to CYP2C19 enzymatic activity and interindividual variability in VCZ plasma concentrations (Hicks et al., 2014). CYP2C19 Ultrarapid or Rapid Metabolizers (UM or RM) phenotypes have been related to lower VCZ plasma concentrations than Normal Metabolizers (NM) and Intermediate or Poor Metabolizers (IM or PM) to higher VCZ plasma concentrations. In this context, *CYP2C19* genotyping for CYP2C19 phenotype inference represents a good tool for the individualization of VCZ therapy. Moreover, the Clinical Pharmacogenetics Implementation Consortium (CPIC) (Moriyama et al., 2017) and the Royal Dutch Association for the Advancement of Pharmacy Pharmacogenetics Working Group (DPWG) (Swen et al., 2011) have developed clinical guidelines for VCZ dose adjustment based on *CYP2C19* genotype. Up to 35 variant star (*) alleles along the *CYP2C19* gene have been described by the Pharmacogene Variation (PharmVar) Consortium (www.PharmVar.org) related to absent, reduced, or increased enzymatic CYP2C19 activities.

Although clinical guidelines for VCZ dose adjustment based on *CYP2C19* genotype could be of enormous help to individualize VCZ treatment, the existing recommendations make no relevant distinction between adult and pediatric patients and are not very specific. CPIC guideline for VCZ and CYP2C19 recommends selecting other antifungal agents in adult and pediatric UM and in adult RM; in PM, they also recommend selecting another antifungal agent, except in those patients in which VCZ is considered to be the most appropriate treatment, where they propose a preferably lower than standard dosage with therapeutic drug monitoring (TDM) (Moriyama et al., 2017). The DPWG suggests a dose adjustment for UM and PM but does not differentiate between adults and children (Swen et al., 2011).

In 2014, Hicks et al. performed a retrospective review focusing on immunocompromised patients with cancer prescribed VCZ for either antifungal prophylaxis or treatment of an IFI at the St Jude Children’s Research Hospital in order to describe the association between *CYP2C19* genotype and VCZ trough concentrations. In those patients carrying the CYP2C19*17 allele, related to increased enzymatic activity, the number of patients achieving VCZ target concentrations was lower than in the other CYP2C19 phenotypic groups. In contrast, VCZ plasma concentrations in those patients carrying CYP2C19*2 allele, related to decreased enzymatic activity, were generally higher. Taking into account these observations, this group developed a second approach consisting of the calculation of an extrapolated VCZ daily dose for each *CYP2C19* group that would allow increasing the number of patients achieving the VCZ therapeutic range. This study proposed that dose modifications based on pharmacogenetic (PGx) information could be an interesting tool for VCZ therapy optimization and individualization.

In addition, due to its nonlinear pharmacokinetics (PK), some studies recommend routine VCZ TDM as a useful strategy to increase the number of patients that achieve therapeutic plasma concentrations and therefore increase VCZ efficacy and safety. Generally, VCZ though the therapeutic range is set between 1 and 5.5 or 6 μg/ml measured in the first 5 days after drug administration and regularly thereafter (Ashbee et al., 2014; Boast et al., 2016; Moriyama et al., 2017).

Despite the fact that TDM is of great help to achieve therapeutic levels, initial low plasma concentration may increase the risk of mortality, even if TDM is subsequently used to achieve target concentrations (Park et al., 2012). There is evidence showing that obtaining VCZ therapeutic levels in the first week of treatment is related to the clinical outcome of fungal infection, as well as to the tolerability to the treatment, decreasing the dose-dependent adverse effects (Ashbee et al., 2014).

**FIGURE 1:**
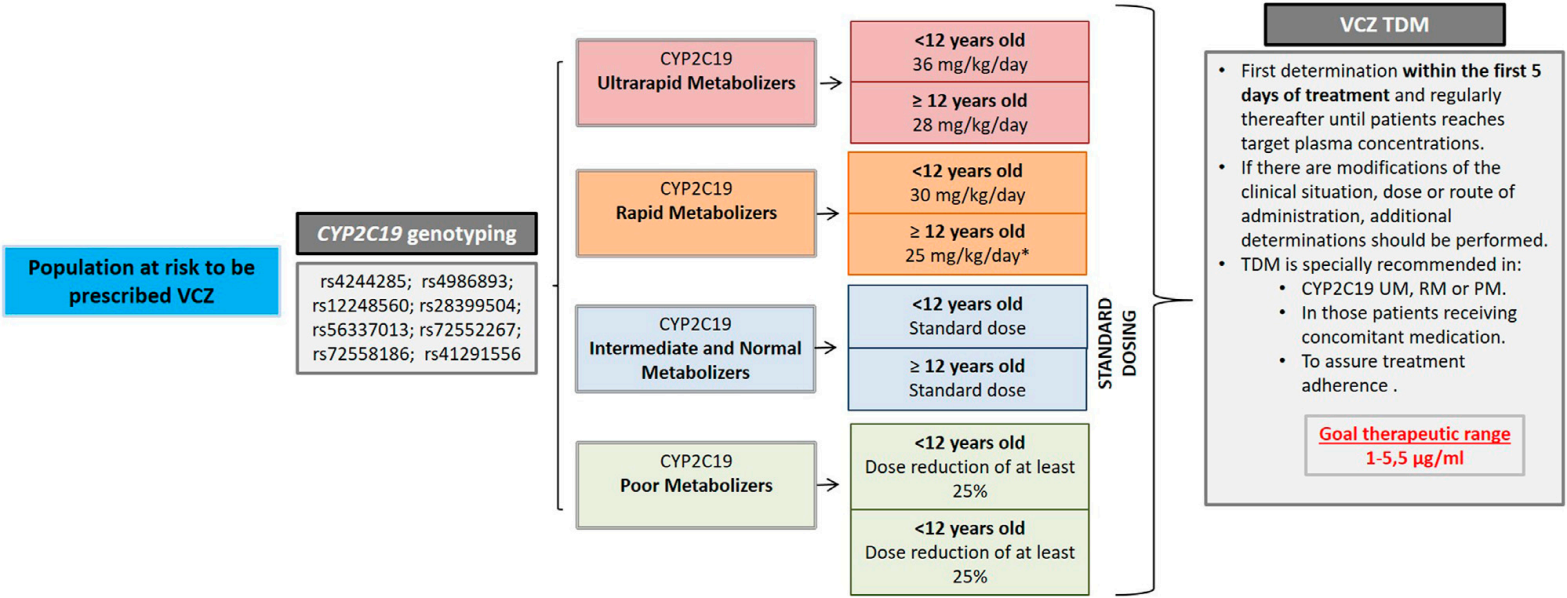
Strategy implemented in La Paz University Hospital (HULP) for the individualization of VCZ therapy in 2017. Dose adjustments based on PGx information were derived from Hicks et al.’s study (Hicks et al., 2014) and CPIC guidelines for CYP2C19 and VCZ therapy (Moriyama et al., 2017) except dose adjustment for Rapid Metabolizer (RM) patients ≥12 years old (*), which were based on literature, and observations made in our hospital Hicks et al. (2014) proposed standard dosing for these patients. For poor Metabolizers, strict controls every 24 h are recommended, as the risk of toxicity is elevated. If ADRs are detected, choose an alternative agent. TDM recommendations were based on TDM guidelines of the British Society for Medical Mycology (Ashbee et al., 2014).

Taking into account the reported studies, in 2017, we included VCZ in our strategy for the implementation of pharmacogenetics in our hospital (Luong et al., 2016a). This strategy is framed within the Clinical Pharmacogenetics Unit of La Paz University Hospital (HULP) and therefore a clinical protocol was developed in collaboration with the Pediatric Oncology and Hematology Department for individualization of VCZ therapy in immunosuppressed patients. Our strategy consisted of a combination of both preemptive genotyping of *CYP2C19* (for optimizing initial dosing) and routine TDM in hematological patients undergoing allogeneic hematopoietic stem cell transplantation (alloHSCT) with a high risk of developing IFI and who will receive VCZ as either prophylaxis or treatment (Figure 1).

We aimed to provide information about our experience implementing a strategy to individualize VCZ treatment including *CYP2C19* preemptive genotyping and TDM in immunocompromised pediatric patients. Also, our objective is to compare our results with the standard care results obtained by Hicks et al. and those expected in their simulation of VCZ dosing based on PGx (Hicks et al., 2014) as a measure to evaluate its efficacy for (a) incrementing the number of patients within therapeutic range 5 days after VCZ administration and (b) reducing the time required to achieve therapeutic plasma concentrations during treatment/prophylaxis.

## Methods

### Patients and Study Design

The study was designed as a single-center, retrospective study, focusing on immunocompromised pediatric patients. The patients selected were managed according to the protocol described in Figure 1 that was implemented in routine care in 2017, prior to conducting this study. Patients genotyped for *CYP2C19* that eventually received VCZ and had at least one VCZ plasma trough concentration were eligible for this study. We selected a cohort of 28 immunocompromised patients with malignant conditions undergoing alloHSCT who were prescribed VCZ either as prophylaxis or treatment for a suspected IFI. All patients were pediatric, aged 1–18 years. All the participants/their parents or legal guardians (if applicable) provided written consent before the pharmacogenetics study.

According to our protocol, at the first clinical evaluation previous to alloHSCT, blood samples are collected and sent to the Clinical Pharmacogenetics Unit for preemptive genotyping of *CYP2C19*. Therefore, genetic results are available at the time of VCZ prescription through the Electronic Health Record (EHR). An initial sampling of VCZ concentrations is indicated within the first 5 days of treatment. Regular PK measures should be performed thereafter until patients reached target plasma concentrations (1–5.5 μg/ml) or until treatment or prophylaxis is completed (Park et al., 2012; Ashbee et al., 2014; Luong et al., 2016b). Prophylaxis is usually maintained for 100 days, but it can be extended to day 180 in patients with continuous immunosuppression or graft vs. host disease. Treatment is maintained until IFI completes remission.

This study is under the umbrella of a master protocol approved by the Ethics Committee (CEIm) of Hospital Universitario La Paz (Identifier: Clinical Ethical Approval No. PEI-2915) on September 21, 2017.

### Pharmacogenetic Study

Molecular analysis was performed in all 28 patients for the selected SNPs of the *CYP2C19* gene: rs4244285 (c.681G > A), rs4986893 (c.636G > A), rs12248560 (c.−806C > T), rs28399504 (c.1A > G), rs56337013 (c.1297C > T), rs72552267 (c.395G > A), rs72558186 (c.819 + 2 T > A), and rs41291556 (c.358 T > C) using our custom SNP-array platform PharmArray®. Genotypes were codified to the star-allele nomenclature (*) using the Haplotype Set IDs provided by PharmGKB (Whirl-Carrillo et al., 2012) and PhamVar (PA166128323) (Gaedigk et al., 2018). CYP2C19 phenotypes were inferred using the CPIC standardized allele definition and functionality tables (PA166124411) as well as specific clinical guidelines (Moriyama et al., 2016). The final molecular report was integrated in the EHR of each patient.

### VCZ Initial Dose Adjustment Based on PGx Results

Initial dose adjustment recommendations were made by the Clinical Pharmacology Department and were mainly based on Hicks et al. calculations of extrapolated doses (Hicks et al., 2014) and CPIC clinical guidelines (Moriyama et al., 2017). Our individualization strategy included modification of dosage in CYP2C19*1/*17 patients ≥12 years old. These patients were assigned a dose of 14 mg/kg/day in Hicks et al. simulation and pediatric patients with this phenotype were recommended to initiate therapy with standard care dosing by CPIC clinical guidelines; however, there is evidence demonstrating that these patients are likely to have subtherapeutic trough concentrations when receiving standard doses (OwusuObeng et al., 2014; Hamadeh et al., 2017). Based on the literature and our previous experience, we considered that these patients required higher doses to achieve target concentrations and we recommended an initial dose of 25 mg/kg/day instead.

Figure 1 shows our VCZ therapy individualization strategy. Clinical recommendations based on genetic results were incorporated into the EHR.

### Analysis of VCZ Plasma Concentrations and Dose Adjustment Based on TDM

VCZ plasma concentrations were measured at Hospital La Paz in the TDM Laboratory of the Clinical Pharmacology Department by immunoassay analysis: ARK voriconazole assay (Thermo Scientific) in a Dimension^®^ EXL 200 de Siemens^®^. The lower limit of VCZ detection was 0.7 μg/ml and the upper limit of detection was 16.0 μg/ml.

The samples analyzed were trough concentration. Samples were sent to the TDM Laboratory following standard clinical procedure for hospital samples.

Plasma trough concentrations as per our protocol are recommended to be measured within the first 5 days of VCZ administration and regularly thereafter until target concentrations (1–5.5 μg/ml) are reached.

TDM recommendations were based on TDM guidelines of the British Society for Medical Mycology (Ashbee et al., 2014). TDM recommendations were also incorporated into the EHR.

### Statistical Analysis

Descriptive statistics were calculated for all variables, with percentages being reported. The Shapiro Wilks test was used to contrast if the first concentration measure after initial dose adjustment based on PGx was normally distributed in our population. We rejected the null hypothesis in the test for normality (*p* < 0.001) concluding that concentration shows a nonnormal distribution. Statistical analyses were performed using R software (V 3.6.3). To assess for significant differences between phenotype and first concentration measure after initial dose adjustment based on PGx, the Mann–Whitney *U* test was applied.

## Results

### Study Population Characteristics

The demographic and clinical data as well as *CYP2C19* diplotype frequencies found in our cohort are summarized in Table 1. Our population consisted of 28 immunocompromised pediatric patients undergoing allogeneic hematopoietic stem cell transplantation due to different malignancies and therefore at risk of invasive fungal disease. The study population was stratified by age (≤11 years and ≥12 years) to properly compare our results with those in Hicks et al.’s study (Hicks et al., 2014): 79% (*N* = 22) of the patients were 11 years old and younger and 21% (*N* = 6) were over 11 years old. A comparison between *CYP2C19* diplotype frequencies found in our cohort of the Spanish population and those found in Hicks et al.’s study (Hicks et al., 2014) is shown in Table 1. After molecular analysis, we recommended different initial doses of VCZ depending on the CYP2C19 phenotypic classification. The average time from molecular study request to the incorporation of the clinical recommendations into the EHR was 21.9 days. The final pharmacogenetic clinical report was always available at the time of prescription.

**TABLE 1:** Patients characteristics (*N* = 28). Patients characteristics found in our cohort of the Spanish population are compared to those in Hicks et al.’s study (*N* = 33).

			All patients *N* = 28	Hicks et al. *N* = 33
Age	<12 years old, n(%)		22 (78.57%)	19 (58.58%)
	≥12 years old, n(%)		6 (21.43%)	14 (42.42%)
	Median (years) [range (years)]		9.5 [<1–17]	9.0 [1–19]
Gender, n (%)	Female		16 (57.14%)	14 (42.42%)
	Male		12 (42.86%)	19 (58.58%)
*CYP2C19* dyplotype, n (%)	CYP2C19*17/*17		2 (7.14%)	4 (12.12%)
	CYP2C19*1/*17		6 (21.43%)	8 (24.24%)
	CYP2C19*2/*17		3 (10.71%)	0 (0%)
	CYP2C19*1/*1		13 (46.43%)	11 (33.33%)
	CYP2C19*1/*2		4 (14.29%)	9 (27.27%)
	CYP2C19*2/*2		0 (0%)	1 (3.03%)
Primary diagnosis, n (%)	Acute lymphoblastic leukemia		5 (17.9%)	12 (36.4%)
	Acute myeloid leukimia		6 (21.4%)	13 (39.4%)
	Non-Hodgkin lymphoma		1 (3.6%)	3 (9.1%)
	Other		16 (57.1%)	5 (15.1%)
		Severe aplastic anemia posthepatitis	2
		Acute biphenotypic leukemia	1
		Autoimmune lymphoproliferative syndrome due to CTLA4 haploinsufficiency	1
		Fanconi anemia	1
		Idiopathic aplastic anemia	4
		Malignant infantile osteopetrosis	1
		Combined immunodeficiency	4
		Chronic granulomatous disease	1
		Sickle cell anemia	1

Based on the previously mentioned criteria (Figure 1), we found that standard dose modifications were indicated in 29% (*N* = 8) of the patients in our cohort. CYP2C19*1/*1, *1/*2, and *2/*17 patients (CYP2C19 NM and IM) were assigned standard initial doses, whereas CYP2C19*1/*17 and *17/*17 patients (CYP2C19 RM and UM) were recommended increased starting doses (Figure 1). No PM were found in our cohort.

### Pharmacokinetic Evaluation of a Strategy for the Individualization of VCZ Treatment Based on PGx and TDM

The average VCZ trough concentration was 2.15 ± 2.62 μg/ml for all *CYP2C19* diplotype groups. There were no significant differences between groups.

**FIGURE 2:**
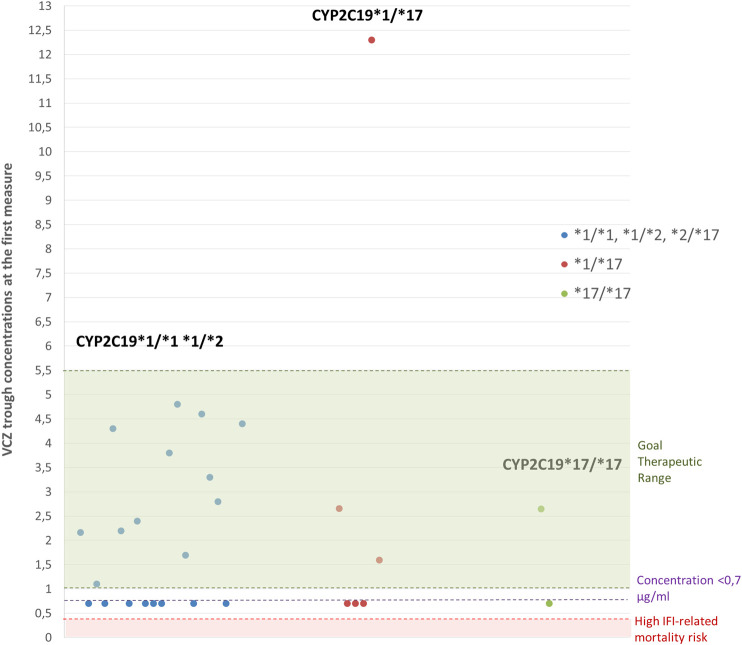
VCZ trough concentrations obtained at the first measure after initial dose adjustment based on PGx. Green area represents target therapeutic range (1–5.5 μg/ml). The red area (<0.35 μg/ml) represents a high-risk area of IFI-related mortality (Miyakis et al., 2010). *N* = 28 patients.

We found that 57.14% (*N* = 16) of the patients achieved target VCZ concentrations in the first VCZ level determination after genetic results were available for initial dose adjustment. When subdividing by CYP2C19 phenotypic group, we can see that 65% (*N* = 13) of the CYP2C19 NM and IM (assigned with standard initial doses), 33.33% (*N* = 2) of the RM, and 50% (*N* = 1) of the UM achieved goal therapeutic range in the first VCZ level determination after genetic results were available for initial dose adjustment. (Table 2.1A). Figure 2 shows VCZ trough concentrations at the first measure after initial dose adjustment based on PGx.

**TABLE 2:** Percentage of patients in the goal therapeutic range (1–5,5 μg/ml) by *CYP2C19* diplotype in our study cohort including 1) all groups of age, 2) patients <12 years old, and 3) patients >12 years old. (A) First trough level measure after initial dose adjustment based on PGx. (B) Level within goal therapeutic range measured after the first trough.

CYP2C19 diplotype classification	CYP2C19 inferred phenotype	(A) % of patients in goal therapeutic range at first measure after initial dose adjustment based on PGx	(B) % of patients in goal therapeutic range at any level extracted after the first trough
**(1) All groups of age (*N* = 28)**		57.14%	92.86%
CYP2C19*1/*1, *1/*2, and *2/*17	CYP2C19 Normal and Intermediate Metabolizers (NM and IM)	65.00%	90%
CYP2C19*1/*17	CYP2C19 Rapid Metabolizers (RM)	33.33%	100%
CYP2C19*17/*17	CYP2C19 Ultrarapid Metabolizers (UM)	50.00%	100%
**(2) Patients <12 years old (*N* = 22)**			
CYP2C19*1/*1, *1/*2, and *2/*17	CYP2C19 NM and IM	62.50%	87.5%
CYP2C19*1/*17	CYP2C19 RM	25.00%	100%
CYP2C19*17/*17	CYP2C19 UM	50.00%	100%
**(3) Patients >12 years old (*N* = 6)**			
CYP2C19*1/*1, *1/*2, and *2/*17	CYP2C19 NM and IM	75.00%	100%
CYP2C19*1/*17	CYP2C19 RM	50.00%	100%
CYP2C19*17/*17	CYP2C19 UM	50.00%	100%

In the subgroup of patients under 12 years old, we found that 62.50% of the CYP2C19 NM and IM, 25% of the RM, and 50% of the UM achieved goal therapeutic range in the first measure after initial dose adjustment based on PGx (Table 2.2A). The number of patients ≥12 years old in our cohort is limited (*N* = 6). In this subgroup of patients, 75% of the CYP2C19 NM and IM, 50% of the RM, and 50% of the UM achieved goal therapeutic range in the first measure after initial dose adjustment based on PGx (Table 2.3A).

**FIGURE 3:**
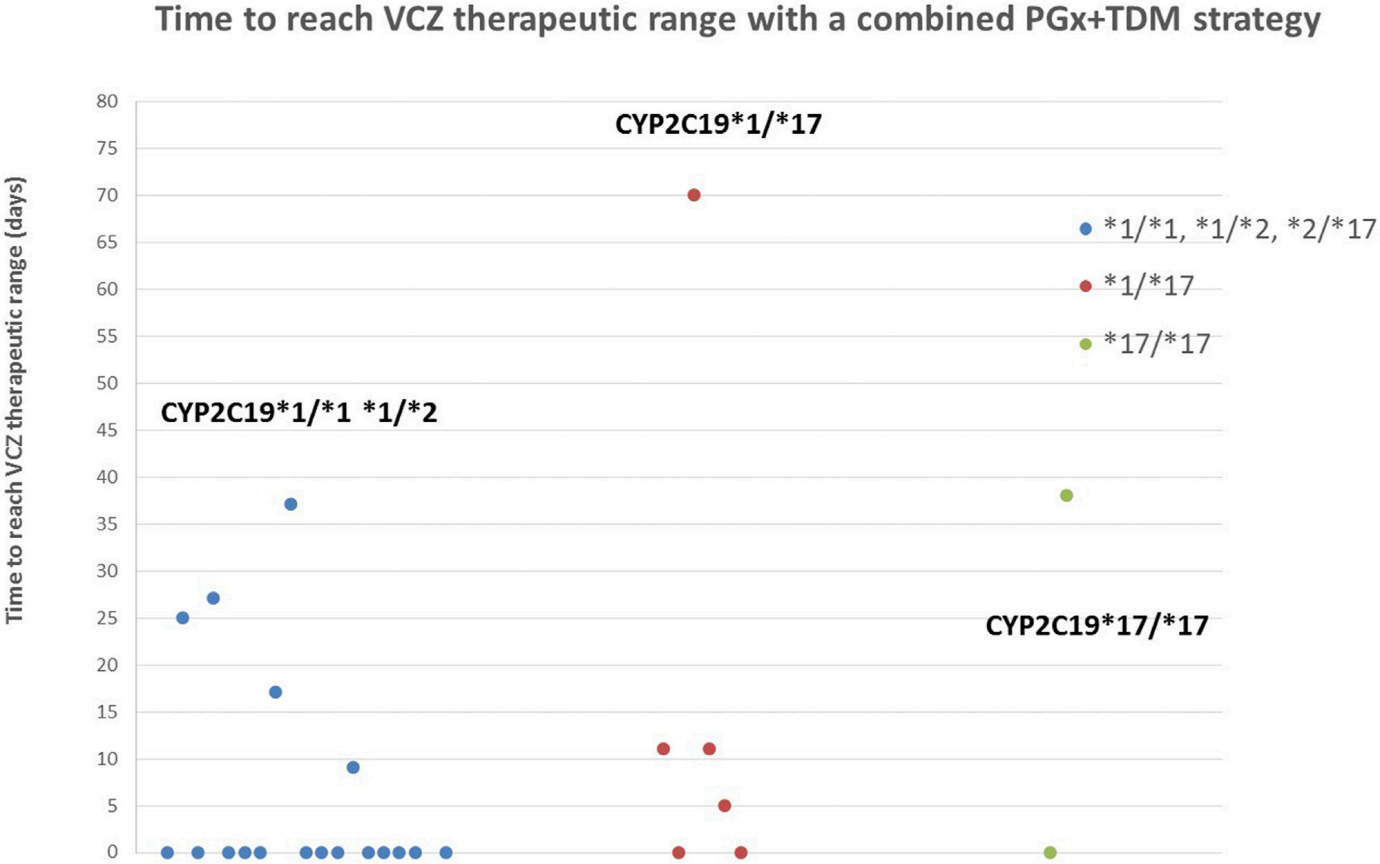
Time to reach VCZ goal therapeutic range (1–5,5 μg/ml) with a combined PGx and TDM strategy. 75% of the patients achieved target concentrations within the first 20 days and 92.85% before the end of treatment/prophylaxis.

In those patients who were not able to achieve target concentrations in the first level measured, VCZ trough concentrations were regularly assessed in order to guide dose modifications and achieve the goal therapeutic range. In our study including all groups of age, we found that 90% of the CYP2C19 NM and IM and 100% of the CYP2C19 RM and UM achieved target concentrations during treatment/prophylaxis (Table 2.1B). The observed times required to achieve the goal therapeutic range for each patient are represented in Figure 3; 75% (*N* = 21) of the patients achieved target concentrations within the first 20 days of treatment.

## Discussion

Due to its great interindividual variability in plasma concentrations and clinical response, there is a growing interest in personalizing VCZ therapeutic strategies for each patient. To this aim, optimization of VCZ initial dosing and TDM have been reported as interesting tools for guiding VCZ treatment and prophylaxis (Park et al., 2012; Ashbee et al., 2014; Hicks et al., 2014; Boast et al., 2016). In this context, our group has designed a protocol for the individualization of VCZ therapy in immunocompromised patients pre-alloHSCT based on PGx (for the optimization of initial dosing) and routine TDM for further dose adjustments. We implemented a “preemptive genotyping strategy in a predefined risk population” (Luong et al., 2016a), where molecular analysis was requested in the first clinical evaluations pre-alloHSCT. Therefore, short response times were required, so molecular and clinical reports could be available at the time of VCZ prescription. The average response time in our study cohort was 21.9 days and met the required treating deadlines.

Taking into account molecular results and based on Hicks et al. simulation (Hicks et al., 2014), CPIC clinical guidelines, and previous own experience, we recommended modifications of initial standard dosing in 29% of the patients. We found that 57.14% of our patients achieved target VCZ concentrations in the first measure after initial dose adjustment based on PGx. In contrast, only 46.5% of VCZ troughs (obtained at a steady state) from the patients in Hicks et al.’s study, where all patients were treated with standard VCZ regimens, were within the therapeutic range (Hicks et al., 2014). In their simulation with extrapolated initial doses, Hicks et al. predicted that 60% of the VCZ troughs would be within the therapeutic range, similar to the results in our cohort. Results of our cohort stratified by phenotype and age can be found in Table 2. Table 3 shows a comparison between our overall results and those found in Hicks et al.’s study. Table 4 shows a comparison between our results and those found in Hicks et al.’s study subgrouped by phenotype.

**TABLE 3:** Percentage of patients^a^/troughs^b^ within goal therapeutic range. Comparison of our results (A) with those found in Hicks et al.’s study with standard care and (B) simulation with extrapolated doses (C).

Study Cohort (*N* = 28)	Hicks et al. (*N* = 33)	
(A) % of patients in goal therapeutic range: first measure after initial dose adjustment based on PGx	(B) % of voriconazole troughs within the goal therapeutic range (Hicks et al. standard care)	(C) % of voriconazole troughs within the goal therapeutic range (Hicks et al. simulation with extrapolated doses)
57.14%	46.5%	60%

a In our study, the percentage of patients within the goal therapeutic range were calculated.

b Hicks et al. calculated the proportion of voriconazole troughs within the therapeutic range. The proportion of patients within the goal therapeutic range could not be extracted from Hicks et al.’s data.

**TABLE 4:** Percentage of patients^a^/troughs^b^ within goal therapeutic range subgrouped by phenotype. Comparison of our results (A) with those found in Hicks et al.’s study with standard care (B). All groups of age.

	(A) Study cohort (*N* = 28)	(B) Hicks et al. (*N* = 33)
CYP2C19 inferred phenotype	% of patients in goal therapeutic range: first measure after initial dose adjustment based on PGx	% of voriconazole troughs within the goal therapeutic range^c^ (Hicks et al.)
CYP2C19 NM	69.23%	63.66%
CYP2C19 IM/Poor Metabolizers (PM)	57.14^e^	90%^d^
CYP2C19 RM	33.33%	50%
CYP2C19 UM	50.00%	0%

a In our study, the percentage of patients within the goal therapeutic range was calculated.

b Hicks et al. calculated the proportion of voriconazole troughs within the therapeutic range. The proportion of patients within the goal therapeutic range could not be extracted from Hicks et al.’s data.

c Voriconazole trough concentrations are the mean voriconazole thought concentrations per patient obtained from a scatter plot from Hicks et al.’s manuscript (Hicks et al., 2014).

d This group in Hicks et al.’s study included Intermediate and Poor Metabolizers (IM and PM) (CYP2C19*1/*2A, CYP2C19*1/*2B, and CYP2C19*2A/*2A).

e This group in our study included only IM (CYP2C19*1/*2 and CYP2C19*1/*17). No PM were found in our study.

In our cohort, CYP2C19*1/*1, *1/*2, and *2/*17 (CYP2C19 NM and IM) were assigned standard initial doses, resulting in 65% of the patients achieving target concentrations in the first trough concentration determination. CYP2C19*1/*17 patients were recommended an initial standard dose of 25 mg/kg/day in patients ≥12 years old and 30 mg/kg/day in younger patients resulting in 33.33% of the patients achieving target concentrations in the first 5 days (Table 2.1A). Hicks et al. reported that only 21% of the troughs in of RM < 12 years old were within concentration range with standard care (Hicks et al., 2014) (Table 5.1B). In our cohort (where 79% of the patients were <12 years old), guiding initial doses based on *P*Gx information increased the percentage of RM achieving VCZ therapeutic range: 25% of RM < 12 (Table 5.1A). Hicks et al. did not propose dose modifications for RM patients ≥12 years old and predicted that up to 57% of the patients could achieve therapeutic range with standard doses (Hicks et al., 2014). However, based on our previous clinical experience, we recommended an increase of standard initial doses also in older patients (25 mg/kg/day) resulting in 50% of RM ≥ 12 years old achieving target concentrations (Table 5.2A).

**TABLE 5:** Percentage of patients^a^/troughs^b^ within goal therapeutic range subgrouped by phenotype and age. Comparison of our results (A) with those found in Hicks et al.’s study with standard care and (B) and simulation with extrapolated doses (C).

		Study cohort	Hicks et al.	
*CYP2C19* diplotype classification	CYP2C19 inferred phenotype	(A) % of patients in goal therapeutic range: first measure after initial dose adjustment based on PGx	(B) % of voriconazole troughs within the goal therapeutic range (Hicks et al. standard care)	(C) % of voriconazole troughs within the goal therapeutic range (Hicks et al. simulation with extrapolated doses)
**Patients <12 years old (1)**		**(*N* = 22)**	**(*N* = 19)**	
CYP2C19*1/*1	CYP2C19 NM	66.67%	51%	54%
CYP2C19*1/*2	CYP2C19 IM	100%	65%	88%
CYP2C19*2/*17	CYP2C19 IM	33.33%	NA	NA
CYP2C19*1/*17	CYP2C19 RM^a^	25.00%	21%	52%
CYP2C19*17/*17	CYP2C19 UM^a^	50.00%	0%	50%
**Patients ≥12 years old (2)**		**(*N* = 6)**	**(*N* = 13)**	
CYP2C19*1/*1	CYP2C19 NM	50%	36%	36%
CYP2C19*1/*2	CYP2C19 IM	66.67%	63%	63%
CYP2C19*2/*17	CYP2C19 IM	NA	NA	NA
CYP2C19*1/*17	CYP2C19 RM	50%	57%	57%
CYP2C19*17/*17	CYP2C19 UM	NA	0%	100%

a In our study, the percentage of patients within the goal therapeutic range was calculated.

b Hicks et al. calculated the proportion of voriconazole troughs within the therapeutic range. The proportion of patients within the goal therapeutic range could not be extracted from Hicks et al.’s data.

NA: no data available. No patient with that phenotype was found in that cohort.

As we show in pediatric patients, papers by Hicks et al. (2020) and Patel et al. (2020) reporting adult data demonstrate that increased VCZ dosage in RM/UM leads to a drastic reduction of subtherapeutic concentrations^,^ in adult patients with neutropenic acute myeloid leukemia (Hicks et al., 2020) and in prophylaxis after allogeneic hematopoietic cell transplant (Patel et al., 2020). In this context, we propose that CYP2C19 RM (and UM) dosing recommendations should be reviewed for a greater increase of the percentage of patients achieving goal therapeutic range, still low especially among younger patients.

Finally, PGx-guided initial dosing in our cohort allowed one of the CYP2C19*17/*17 patients to achieve VCZ therapeutic concentrations in the first measure after VCZ administration. The other UM patient had a first VCZ trough concentration of 0.7 μg/ml and eventually achieved target concentrations after 38 days thanks to TDM (Figure 3). In Hicks et al.’s study, all CYP2C19*17/*17 patients showed subtherapeutic concentrations and contrary to our cohort, none of them achieve concentrations within the therapeutic range. All UM patients in our cohort were under 12 years old (Tables 5.1A, B). Dose adjustments based on preemptive genotyping improved the percentage of patients carrying the CYP2C19*17 achieving promptly target concentrations; however, as mentioned before, dosing recommendations in this population should be reviewed for greater results. Previous studies have reported that supratherapeutic concentrations (>5.5 μg/ml) can be related to the occurrence of adverse effects, especially neurotoxicity (Miyakis et al., 2010; Park et al., 2012). In the first measures after VCZ administration, we only found one CYP2C19 RM patient <12 years old with a VCZ trough concentration of 12 μg/ml (Figure 2). This patient developed voriconazole-induced phototoxicity. However, this was rapidly corrected after TDM and therapeutic VCZ concentrations were achieved in 5 days. This could be due to the presence of drug interactions, nonlinear PK unpredicted variability or a rare *CYP2C19* variant, or genetic variations in other genes involved in the metabolic pathway not detected by our genotyping panel. However, the group of patients more likely to show VCZ plasma concentrations >5.5 μg/ml are CYP2C19*2*/*2 patients, with no representation in this study.

The second tool used in our individualization strategy was TDM for guiding VCZ dose adjustments in those patients that did not achieve therapeutic range in the first measure since VCZ administration. Table 3 shows the percentage of patients that achieve the VCZ therapeutic range before the end of treatment/prophylaxis. We found that 90% of the CYP2C19 NM and IM and 100% of the CYP2C19 RM and UM achieved target concentrations during treatment/prophylaxis and therefore the potential of this strategy to improve dose adjustment. Treatment failure has been reported to occur within the first 35 days of treatment (Miyakis et al., 2010). Implementing our combined *P*Gx and TDM strategy, 75% of the patients achieved target concentrations within the first 20 days of treatment.

The main limitation of our study is that the data were collected retrospectively from medical records and some information was not available. However, data concerning genetic results, dose recommendations based on phenotype, and at least one VCZ plasma trough level were available for all the patients. According to our clinical protocol, an initial sampling of VCZ concentration should be obtained within the first 5 days of treatment; however, not all the clinicians followed this recommendation. Another limitation is that, in those patients who did not achieve therapeutic range in the first measure since VCZ administration, dose adjustment recommendations were based on VCZ trough concentrations; however, final dose modifications were performed at the discretion of the treating specialist. Also, another limitation of the study is the lack of a control group in which dose adjustments are based only on TDM. Finally, probably due to relatively small sample size, PM were not represented in our cohort; in spite of this, our protocol includes dose recommendations for these patients: dose reduction of at least 25% of standard dose and early and strict TDM to minimize the risk of concentrations above the therapeutic range (Scholz et al., 2009; Lee et al., 2012; Wang et al., 2014b; Hicks et al., 2014). Despite the fact that our study is not exempt from limitations, it provides relevant information about VCZ individualization based on PGx.

Herein, we have described our strategy for VCZ individualization based on PGx and TDM. Unfortunately, the implementation of similar strategies in the clinical practice still faces different challenges: lack of large population-based studies, insufficiency of cost-effectiveness evidence, and the general barriers to pharmacogenetics implementation.

In this context, we are developing a multicenter, randomized clinical trial to evaluate the effectiveness and efficiency of a preemptive genotyping strategy for VCZ, including an economic evaluation from the perspective of the Spanish National Health System. (Lee et al., 2012; MonserratVillatoro et al., 2020).

## Conclusion

Taking into account these results, we can see that there is a need to improve VCZ dose predictions and that PGx represents a helpful tool for initial dose adjustment and optimization, especially in patients with extreme phenotypes, as it helps to increase the number of patients within goal therapeutic range and decreases the time required to achieve target concentrations when compared with standard care. However, due to the VCZ nonlinear pharmacokinetics resulting in unpredictable and unanticipated changes in drug exposure, TDM is extremely important for guiding dose modifications over treatment and prophylaxis. In our experience, a combination of both strategies can be of great benefit for the patients.

## Data Availability

The original contributions presented in the study are included in the article/supplementary materials; further inquiries can be directed to the corresponding author/s.
